# A novel PAD4/SOX4/PU.1 signaling pathway is involved in the committed differentiation of acute promyelocytic leukemia cells into granulocytic cells

**DOI:** 10.18632/oncotarget.6551

**Published:** 2015-12-10

**Authors:** Guanhua Song, Lulu Shi, Yuqi Guo, Linchang Yu, Lin Wang, Xiaoyu Zhang, Lianlian Li, Yang Han, Xia Ren, Qiang Guo, Kehong Bi, Guosheng Jiang

**Affiliations:** ^1^ Department of Hemato-Oncology, Institute of Basic Medicine, Shandong Academy of Medical Sciences, Key Medical Laboratory for Tumor Immunology and Traditional Chinese Medicine Immunology of Shandong, Jinan, Shandong, China; ^2^ Research Center for Medicinal Biotechnology, Shandong Academy of Medicinal Sciences, Jinan, Shandong, China; ^3^ Qianfoshan Hospital of Shandong, Jinan, Shandong, China

**Keywords:** PADI4, methylation, differentiation, leukemia

## Abstract

All-trans retinoic acid (ATRA) treatment yields cure rates > 80% through proteasomal degradation of the PML-RARα fusion protein that typically promotes acute promyelocytic leukemia (APL). However, recent evidence indicates that ATRA can also promote differentiation of leukemia cells that are PML-RARα negative, such as HL-60 cells. Here, gene expression profiling of HL-60 cells was used to investigate the alternative mechanism of impaired differentiation in APL. The expression of peptidylarginine deiminase 4 (PADI4), encoding PAD4, a protein that post-translationally converts arginine into citrulline, was restored during ATRA-induced differentiation. We further identified that hypermethylation in the PADI4 promoter was associated with its transcriptional repression in HL-60 and NB4 (PML-RARα positive) cells. Functionally, PAD4 translocated into the nucleus upon ATRA exposure and promoted ATRA-mediated differentiation. Mechanistic studies using RNAi knockdown or electroporation-mediated delivery of PADI4, along with chromatin immunoprecipitation, helped identify PU.1 as an indirect target and SOX4 as a direct target of PAD4 regulation. Indeed, PAD4 regulates SOX4-mediated PU.1 expression, and thereby the differentiation process, in a SOX4-dependent manner. Taken together, our results highlight an association between PAD4 and DNA hypermethylation in APL and demonstrate that targeting PAD4 or regulating its downstream effectors may be a promising strategy to control differentiation in the clinic.

## INTRODUCTION

The hematologic malignancy acute promyelocytic leukemia (APL) exhibits a failure of myeloid differentiation [1]. The majority of APL cases are characterized by the fusion of the promyelocytic leukemia protein (PML) to the retinoic acid receptoralpha (RARα) transcription factor, resulting in a block to differentiation and an aberrant selfrenewal of APL cells. Most patients with APL can be induced to complete remission by all-trans retinoic acid (ATRA) treatment through presumed degradation of the PML-RARα fusion protein [2, 3]. Interestingly, however, HL-60 cells, which are PML-RARα negative, can also be induced to differentiate into more mature granulocytes by ATRA [4, 5], indicating that degradation of PML-RARα is not the only molecular mechanism that can induce myeloid differentiation. Consequently, understanding the pathogenesis of APL is vital to identifying novel combined treatment that enhances actual cure rates.

Expression of the Peptidylarginine deiminase 4 (PAD4) was first detected in human myeloid leukemia HL-60 cells after ATRA and 1alpha,25-dihydroxyvitamin D(3)-induced differentiation, and it was observed to regulate hematopoietic progenitor proliferation [6, 7]. As the only isotype out of the five PAD family members to be detected in the nucleus [8], PAD4, encoded by *PADI4*, is part of a transcriptional network that regulates pluripotency [9]. Recently, overexpression of *PADI4* was detected in various tumors [10], demonstrating an ability to promote tumorigenesis by repressing tumor suppressor genes such as *OKL38* [11]. However, upregulation of PAD4 also induces apoptosis of hematopoietic cells [12]. Thus, PAD4 has both tumor suppressor and oncogenic properties, and its activity depends on the cellular context, which further prompted us to clarify its function in myeloid differentiation.

In this study, to explore the mechanism of abnormal differentiation of APL, we investigated the mechanism for the suppression of PAD4 in leukemic cells. Its biological and molecular effects were also studied using *in vitro* experiments. We found that suppression of PAD4 was attributable to its promoter methylation and identified PAD4 as the effector functioning to suppress APL development. Restoration of PAD4 promotes differentiation through chromatin regulation of SOX4 via citrullination, thereby leading to upregulation of PU.1. The data further imply that deregulation of PAD4 may be an alternative mechanism for the impaired differentiation of APL, implicating as a potential target for APL treatment.

## RESULTS

### *PADI4* expression increases steadily during the differentiation of leukemia cells

**Figure 1:**
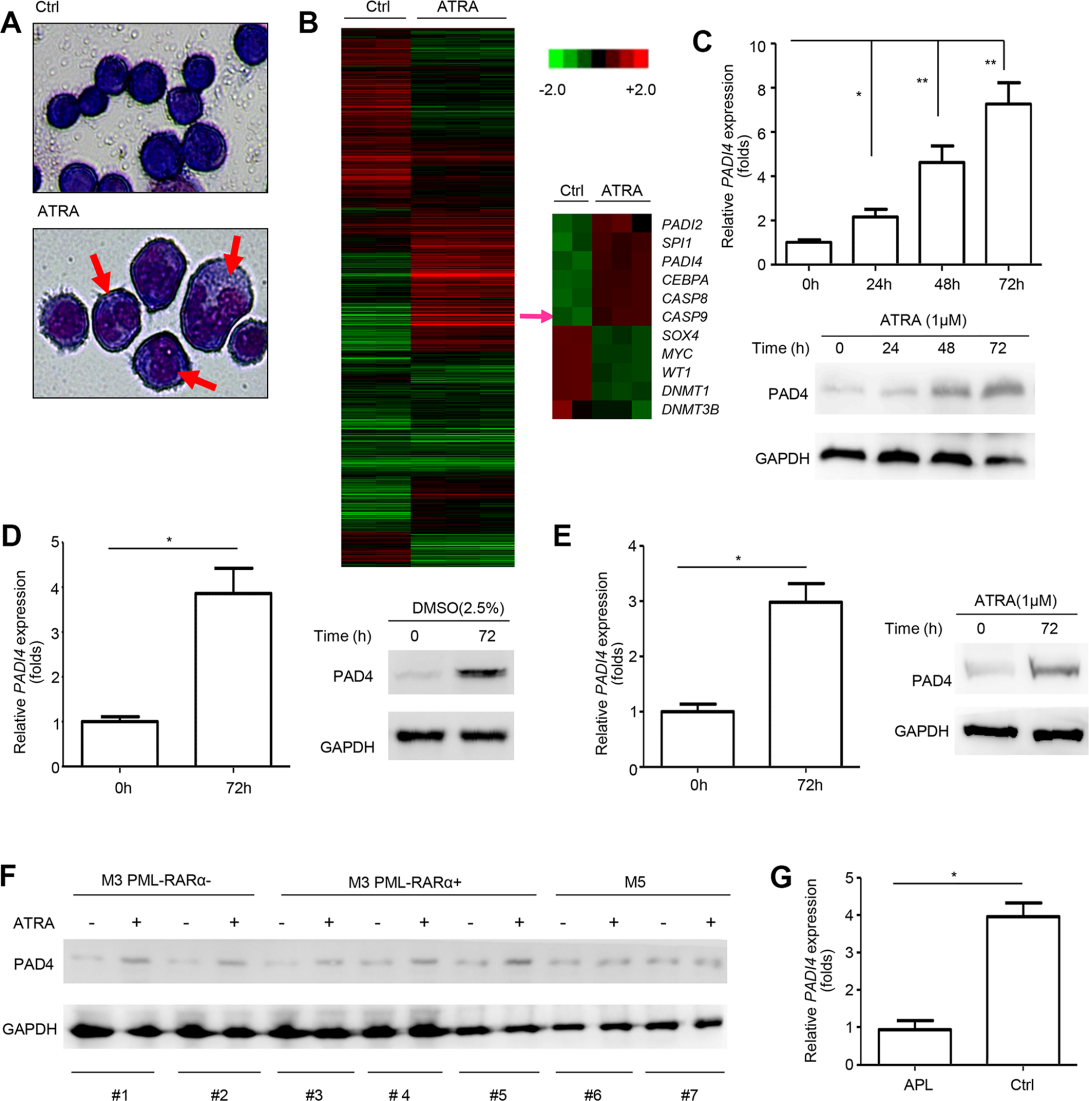
PAD4 expression increased during the differentiation of leukemia cells **(A)** Morphological changes of HL-60 cells after ATRA treatment. **(B)** The cluster heat map shows differently expressed mRNAs in HL-60 before and after ATRA-induced differentiation from microarray data (*P* < *0.05*). **(C)** PAD4 expression was detected at mRNA (top) and protein (bottom) levels after ATRA (1 μM) treatment at various timepoints in HL-60 cells. GAPDH was used to normalize as the loading control. **(D)** PAD4 expression was also assayed in HL-60 cells after DMSO treatment for 72 h by qRT-PCR (left) and Western blot (right). **(E)** qRT-PCR and Western blot were performed to detect the PAD4 expression at mRNA (left) and protein (right) levels in NB4 cells after treatment with ATRA for 72 h. **(F)** The expression of PAD4 was detected by Western blot in clinical samples after ATRA treatment. **(G)** Expression of PAD4 was detected by qRT-PCR in clinical samples of APL (*n* = 12) and the normal controls (*n* = 8). *P < 0.05, **P < 0.01. Data are means of biological triplicates (± standard error) and representative of triplicate experiments.

To systematically study the mechanism underlying abnormal granulocytic differentiation in APL, gene microarray analysis was performed to compare expression profiles in HL-60 cells after ATRA-induced differentiation for 72 hours to those without ATRA treatment. Differentiation was confirmed by morphological changes (Figure 1A). In addition, of genes showing significant changes, many were related to differentiation, such as *SPI1 (PU.1)*, *CEBPA*, *WT1* and *MYC*, or apoptosis, such as *CASP8* (caspase-8) and *CASP9* (caspase-9), further corroborating the validity of our analysis (Figure 1B).

Importantly, among the differentially expressed genes with > 2-fold change, *PADI4* expression increased ∼2.3-fold. Consistent with this, our qRT-PCR and Western blot analysis validated that ATRA stimulation significantly upregulated PAD4 expression at both mRNA (Figure 1C, top) and protein (Figure 1C, bottom) levels in HL-60 cells in a time-dependent manner. In addition, the levels of PAD4 mRNA (Figure 1D, left) and protein (Figure 1D, right) were upregulated by treatment of HL-60 cells with DMSO, which could also promote cell differentiation. Similar results were observed in another leukemia cell line, NB4 (a PML-RARα positive cell line), in which PAD4 mRNA (Figure 1E, left) and protein (Figure 1E, right) levels increased after ATRA treatment.

To exclude the possibility that the induction of PAD4 after ATRA-treatment was caused by non-specific stress, we repeated these experiments in 7 clinical samples in M3 and M5 subtypes, which all belong to acute myeloid leukemia (AML). Western blot results showed that PAD4 expression could only be induced by ATRA treatment in M3 monocytes, both PML-RAR positive and negative, but not in M5 (Figure 1F). Further, the qRT-PCR analysis showed that *PADI4* expression was significantly lower in clinical samples of APL than those of normal controls (Figure 1G). Together, these data indicate that the deregulation of PAD4 expression may be involved in the agonist-induced differentiation of leukemia cells, but not caused by non-specific stress, making it a potential alternative mechanism, besides the fusion of PML and RARα, producing abnormal differentiation in leukemia.

### Methylation in the promoter of *PADI4* depressed its expression in leukemia cells

**Figure 2:**
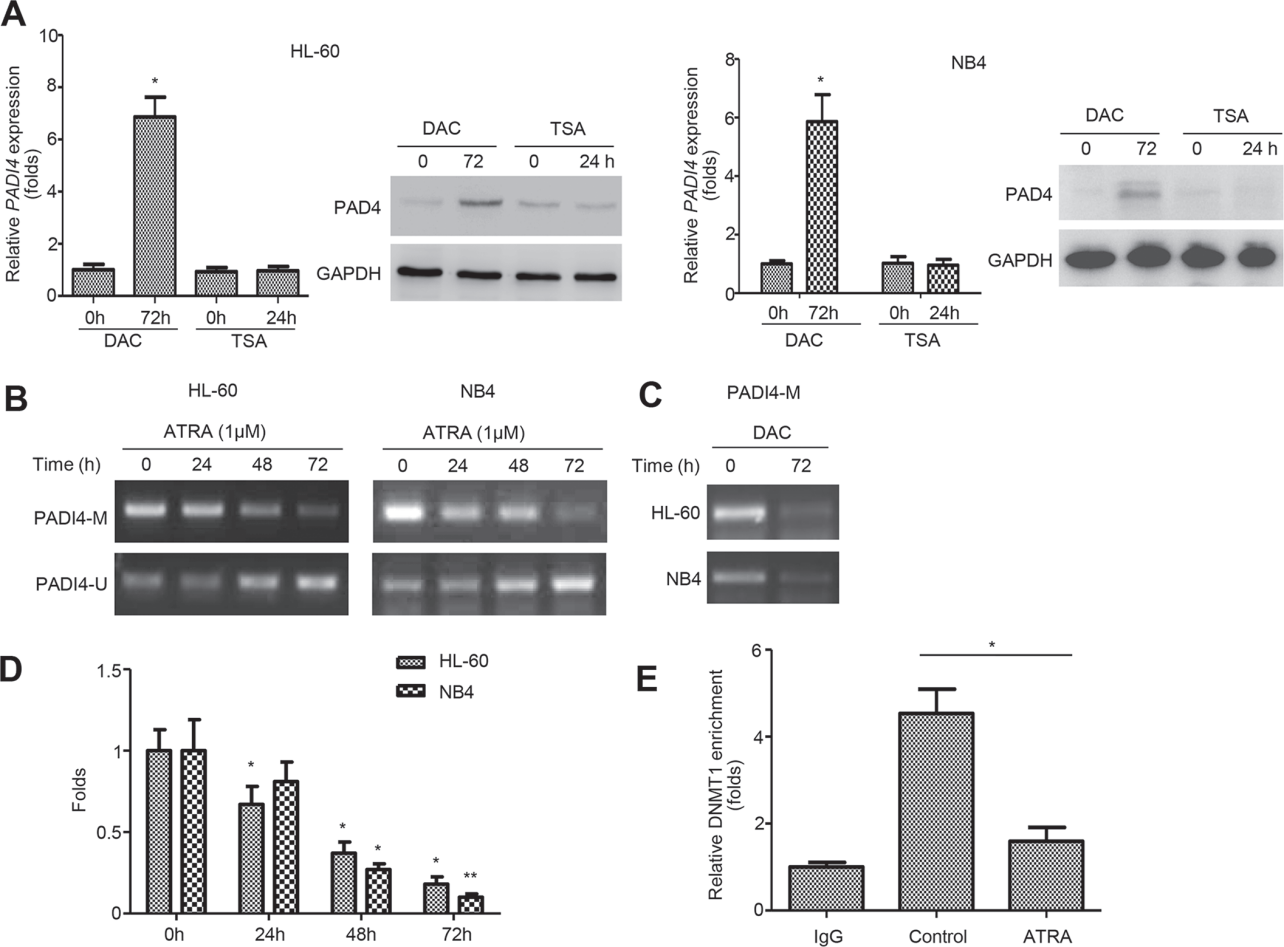
*PADI4* promoter undergoes demethylation during ATRA-induced differentiation **(A)** Expression of PAD4 at mRNA (top) and protein (bottom) levels in HL-60 (left) and NB4 (right) cells before and after treatment with 1.0 mM DAC for 72 h or 200 nM TSA for 24 h. **(B)** MSP was applied to examine the methylation status in the *PADI4* promoter in HL-60 (top) and NB4 (bottom) at indicated timepoints during ATRA-induced differentiation. A PCR band in lane M indicates a methylated *PADI4* gene; a band in lane U indicates an unmethylated *PADI4* gene. **(C)** After DAC treatment for 72 h, changes of DNA methylation in the promoter of *PADI4* was assayed by MSP. **(D)** Relative methylation levels in the *PADI4* promoter region was analyzed with MeDIP-qPCR. **(E)** ChIP analysis was performed to determine the changes of the enrichments of DNMT1 in the *PADI4* promoter. Pre-cleared lysate (1%) was taken before immunoprecipitation and used as the input control. *P < 0.05, **P < 0.01. Data are means of biological triplicates (± standard error) and all data are representative of triplicate experiments.

Epigenetic modifications, such as DNA methylation, regulate gene expression and represent potential targets for differentiation therapy in leukemia [13]. To determine whether methylation regulate PAD4 expression, we incubated HL-60 and NB4 cells with the demethylating agent 5-aza-2′-deoxycytidine (DAC) for 72 h. Treatment with DAC for 72 h effectively restored PAD4 expression at both mRNA (Figure 2A, top) and protein (Figure 2A, bottom) levels in both cell lines, whereas treatment with the histone deacetylase inhibitor trichostatin A (TSA) exerted no visible effects. Methylation-specific PCR (MSP) was then employed and, importantly, the CpG region in the *PADI4* promoter underwent progressive demethylation in a time-dependent manner during ATRA-induced differentiation of HL-60 (Figure 2B, top) and NB4 cells (Figure 2B, bottom). Additionally, DAC treatment resulted in *PADI4* promoter demethylation in both cell lines (Figure 2C). Then, MeDIP analysis was performed using anti-methyl cytosine antibody by immunoprecipitation; the enriched methylated DNA was then used as a template for qPCR amplification of the *PADI4* promoter region containing the CpGs (Figure 2D). ATRA treatment significantly reduced the binding of the enriched *PADI4* promoter containing the CpGs to anti-methyl cytosine antibody. To further support this finding, the expression and activity of DNA methyltransferases (DNMTs) was characterized. Consistent with our microarray data (Figure 1B), the qRT-PCR and Western blot results showed that the expression levels of DNMT1, DNMT3a, and DNMT3b decreased at mRNA (Supplementary Figure 1A) and protein levels (Supplementary Figure 1B) in response to ATRA treatment. Notably, the chromatin immunoprecipitation (ChIP) analysis demonstrated that ATRA treatment suppressed the enrollment of DNMT1 at *PADI4* promoter in HL-60 cells (Figure 2E). These data confirm that methylation is essential for the suppression of PAD4 expression in leukemia cells.

### PAD4 promotes differentiation of leukemia cells and translocates to the nucleus after ATRA-stimulation

**Figure 3:**
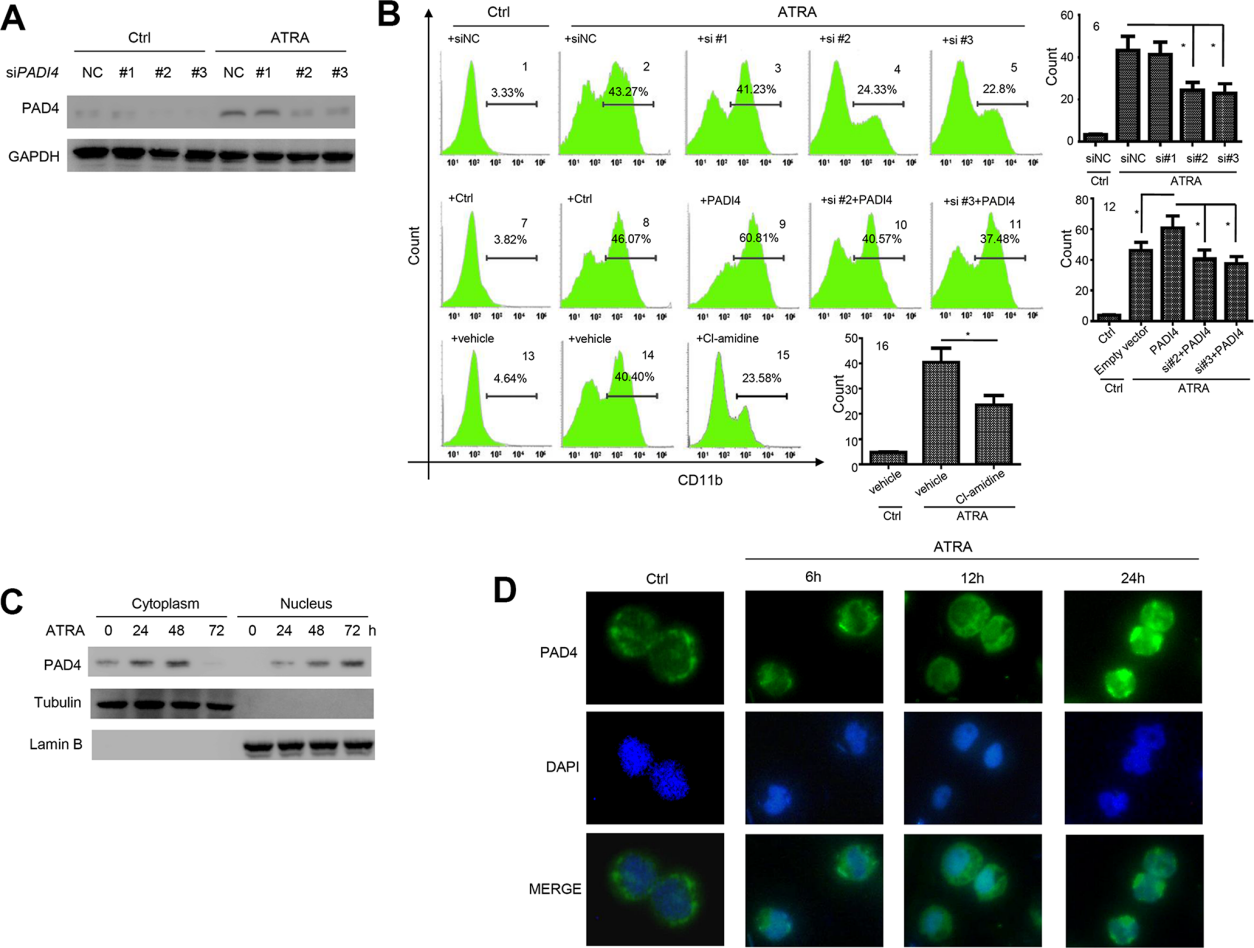
PAD4 promotes leukemia cell differentiation and ATRA treatment facilitates its nucleus translocation **(A)** Efficiency of siRNA transfection targeting PAD4 was validated by Western blot in the absence or presence of ATRA exposure. **(B)** The differentiation of HL-60 cells after silencing **B1–B6** or over-expressing B7–B12 were detected by Flow cytometry. The effect of PAD4 inhibitor, Cl-amidine, on the differentiation of HL-60 cells were also detected by FCM **B13–B16**. *P < 0.05, **P < 0.01. Data are means of biological triplicates (± standard error). (**C**) After treatment with ATRA for various times, the PAD4 expression in cytoplasm (left) and nucleus (right) at protein level was detected by Western blot. Tubulin and Lamin B were applied as loading controls, respectively. **(D)** PAD4 immunostaining (visualized in green) in HL-60 before and after ATRA treatment was assayed by immuno-fluorescent method. All data are representative of triplicate experiments.

To investigate whether PAD4 contributes to the differentiation of leukemia cells, HL-60 cells were pre-transfected with small interfering RNAs (siRNAs) targeting *PADI4* for 6 h, and the silencing efficiency was validated in the presence or absence of ATRA (Figure 3A). Flow cytometry analysis showed that, 1 μM ATRA treatment could induce differentiation of HL-60 (Figure 3B2 and 3B6) when compared with the control (Figure 3B1 and 3B6) as quantified by the synthesis of CD11b, a marker of granulocytic differentiation. However, silencing PAD4 could block the ATRA-induced differentiation of HL-60 cells (Figure 3B4, 3B5 and 3B6) compared with the control (Figure 3B2 and 3B6). Notably, *PADI4* siRNA#1, which had a negligible efficiency to knockdown PAD4, could not block ATRA-induced differentiation (Figure 3B3 and 3B6). In contrast, electroporation-mediated overexpression of PAD4 promoted differentiation of HL-60 cells to a greater extent than ATRA treatment alone (Figure 3B8, 3B9 and 3B12). Furthermore, silencing PAD4 could attenuate the effect of over-expressing it on cell differentiation (Figure 3B10–3B12).

As the enzymatic activity of PAD4 functions mainly through citrullination of its targets [14], we next treated HL-60 cells with the chemical inhibitor Cl-amidine, which disrupts the citrullination activity of PAD4 by introducing a covalent modification in the active site of the enzyme [15]. This treatment blocked ATRA-induced differentiation, as demonstrated by a lower proportion of CD11b-positive cells (Figure 3B15 and 3B16) compared with the vehicle control (Figure 3B14 and 3B16). Thus, PAD4 may promote granulocytic differentiation through post-translational citrullination of its targets.

Western blot analysis demonstrated that PAD4 expression increased over time in both the cytoplasm and nucleus of HL-60 cells following ATRA treatment (Figure 3C). Immunofluorescent staining supported these findings, with the localization of PAD4 predominantly in the nucleus following ATRA treatment (Figure 3D). These results imply that, during differentiation, PAD4 functions predominantly within the nucleus. Therefore, we aimed to investigate the primary target of PADI4 during ATRA-mediated differentiation.

### PAD4 is involved in the regulation of PU.1

**Figure 4:**
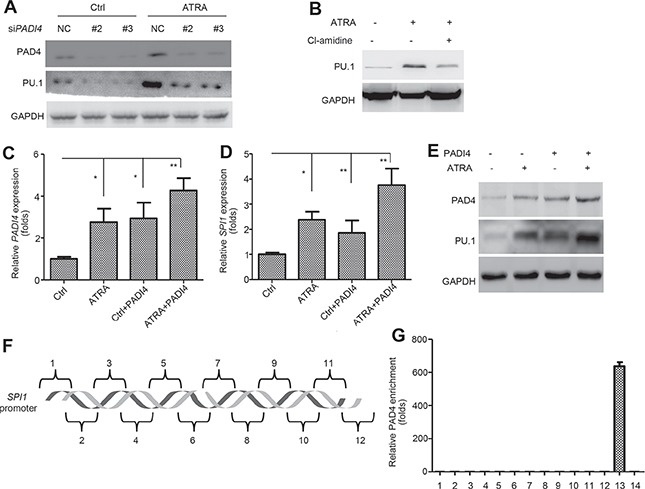
PAD4 indirectly regulates PU.1 during differentiation process **(A)** and **(B)** Effect of silencing PAD4 A or its inhibitor, Cl-amidine B on the PU.1 expression was assayed by Western blot during ATRA-induced differentiation. **(C–E)** The expression of PAD4 and PU.1 was examined by qRT-PCR and Western blot after electroporation-induced PAD4 overexpression in HL-60 with or without ATRA treatment. **(F)** Different regions (1–12) of the *SPI1* promoter are depicted. **(G)** ChIP-qPCR detection of the binding of PAD4 to the *SPI1* promoter in the ChIP assay. 13 indicates the positive control, which was a known PAD4 binding site of *p21*. 14 represents IgG as the negative control. *P < 0.05, **P < 0.01. Data are representative of triplicate experiments.

To further study the pathological mechanism of PAD4 in ATRA-induced differentiation, we explored the potential pathways through which it functions. Several key factors in APL were selected, such as *HOXB6, HOXA9, ERG, WT1, EVI1, CEBPA, MYC* and *SPI1* [16–23]. *SPI1* showed the most significant suppression after silencing PAD4 in ATRA-induced HL-60 (Supplementary Figure 2), indicating that the pathway mediated by PU.1 may contribute to the function of PAD4. The transcription factor PU.1 functions in hematopoietic development, and reduced PU.1 expression is thought to promote the accumulation of immature granulocytic progenitors [21, 24]. In accordance, our microarray data detected higher expression of *SPI1* after ATRA treatment of HL-60 cells (Figure 1B). Indeed, PU.1 was down-regulated after silencing PAD4 in HL-60 cells in the absence (Figure 4A, left) or presence (Figure 4A, right) of ATRA exposure. Accordingly, Cl-amidine treatment also suppressed PU.1 expression (Figure 4B). To further support this, electroporation was used to induce PAD4 expression under ATRA treatment (Figure 4C). Of note, combined treatment of ectopic overexpression of PAD4 and ATRA provoked a more robust induction of PU.1 at mRNA (Figure 4D) and protein (Figure 4E) levels in HL-60 cells than either one alone. Next, ChIP analysis was performed by targeting various regions encompassing −2000 to +20 bp in the promoter of *SPI1* (Figure 4F), and the binding of PAD4 to the promoter of *p21* was treated as the positive control [25]. However, no direct binding of PAD4 was detected in the promoter of *SPI1* (Figure 4G), which indicates an indirect regulation of PAD4 towards PU.1.

### SOX4 is a direct target for PAD4-mediated effects

**Figure 5:**
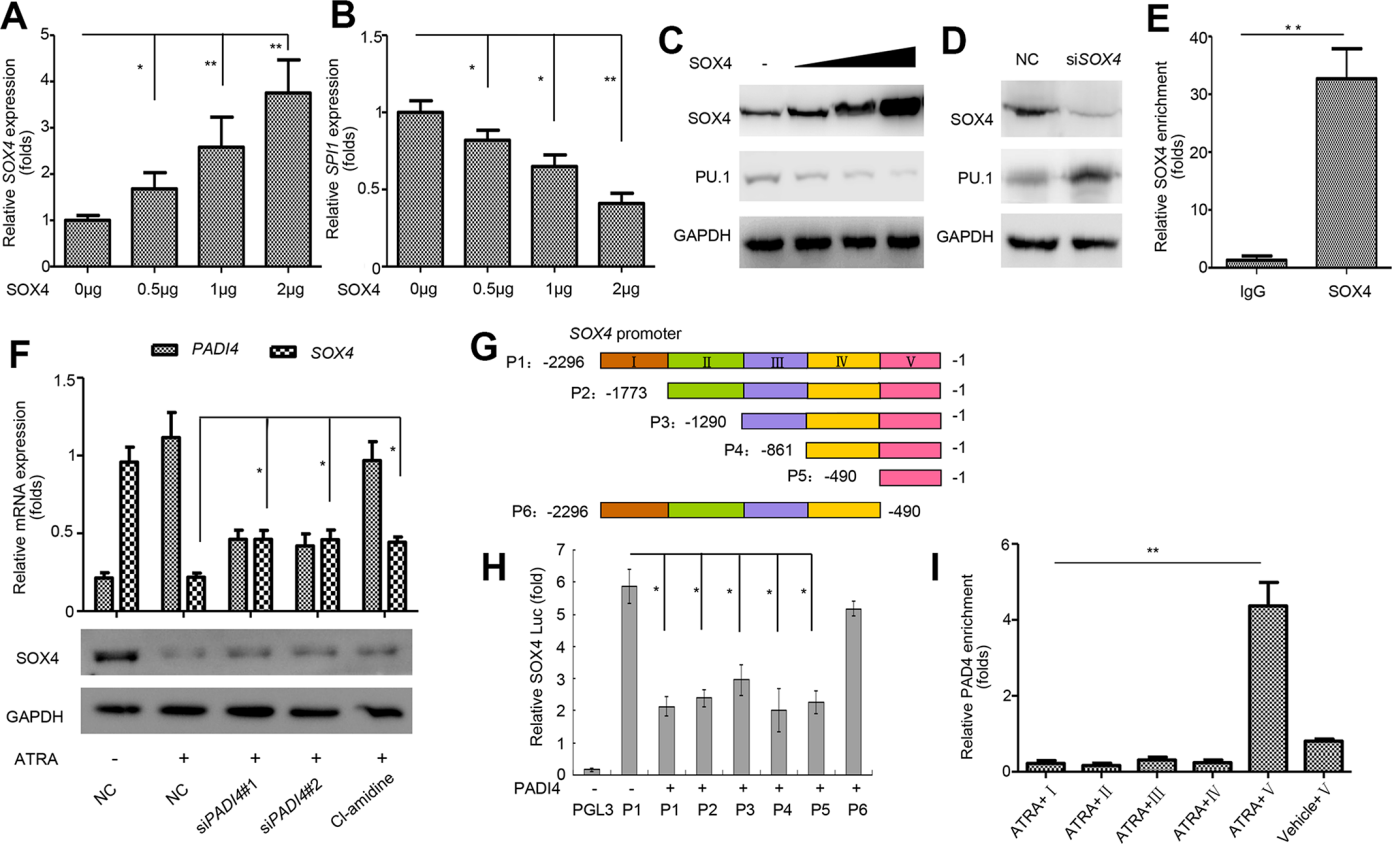
SOX4 mediates the regulation of PU.1 by PAD4 **(A–C)**. qRT-PCRand Western blot were performed to detect the expression of SOX4 and PU.1 in HL-60 cells after overexpressing SOX4 in a gradually increasing manner mediated by electroporation for 36 h. **(D)** Expression of PU.1 in HL-60 cells with siRNA-mediated SOX4 knockdown. **(E)** The binding of SOX4 to *SPI1* promoter was analyzed by ChIP. **(F)** Expression of SOX4 was determined after indicated treatment and GAPDH was applied as the loading control. (**G**) Schematic representation of the promoter region of *SOX4*. P1–P6 represents different regions of *SOX4* promoter as shown in the color map above. **(H)** The pGL3-basic control or various *SOX4* constructs were co-transfected with the PADI4 plasmid into HEK293 cells. 24 hours after transfection, cells were harvested for the luciferase reporter assay. **(I)** ChIP-qPCR was applied to detect the enrichment of PADI4 on the *SOX4* promoter. Upon ChIP with an anti-PADI4 antibody, PCR with primers at different positions of the *SOX4* promoter were performed. I, II, III, IV and V represent various primers amplifying regions of different colors as indicated in the map **(H)**. *P < 0.05, **P < 0.01. Data are means of biological triplicates (± standard error) and representative of triplicate experiments.

PU.1 was previously validated to be directly regulated by RUNX1, HSF-1, NF-κB, STAT3, and SOX4 [26–30]. Our qRT-PCR analysis showed that, in electroporation-mediated overexpressing PAD4 HL-60 cells, *SOX4* was significantly reduced (Supplementary Figure 3), which indicates SOX4 may mediate the regulation of PAD4 towards PU.1. A previous study reported that SOX4 could directly repress PU.1 expression, resulting in the blocked differentiation of myeloid leukemia cells in mouse models [30]. The same regulation was echoed in HL-60 cells as evidenced by the fact that ectopic expression of SOX4 at different concentrations (Figure 5A and 5C) resulted in the down-regulation of PU.1 mRNA (Figure 5B) and protein (Figure 5C) in a dose-dependent manner. In contrast, silencing SOX4 restored the expression of PU.1 (Figure 5D). A ChIP analysis subsequently confirmed that SOX4 could bind to the *SPI1* promoter (Figure 5E).

As *SOX4* expression was depressed following ATRA-induced differentiation in our microarray analysis, we further investigated whether SOX4 is targeted by PAD4. First, silencing PAD4 or Cl-amidine treatment in ATRA-treated HL-60 cells restored SOX4 expression at mRNA and protein levels (Figure 5F). Next, various luciferase reporters were constructed with different regions of the *SOX4* promoter inserted (Figure 5G); these were transiently transfected into HEK293 cells. Ectopic expression of PAD4 reduced pGL3-SOX4 luciferase activity, while the deletion construct (P6) encompassing its promoter region from −490 to +1 attenuated this negative effect (Figure 5H), which indicates that the region P5 (−490 to +1) contains the response elements required for the regulation of SOX4 by PAD4. ChIP assays were performed to further confirm the interaction between PAD4 and the *SOX4* promoter in HL-60 cells. Following ATRA treatment, the specific binding of PAD4 was detected at the *SOX4* promoter fragment between the −490 to +1 bp proximal to the transcription start site (Figure 5I). However, no visible binding was detected in this region in the absence of ATRA, which confirms that SOX4 is a novel target of PAD4 (Figure 5I).

### PAD4 regulates *SOX4* expression through chromatin modification

**Figure 6:**
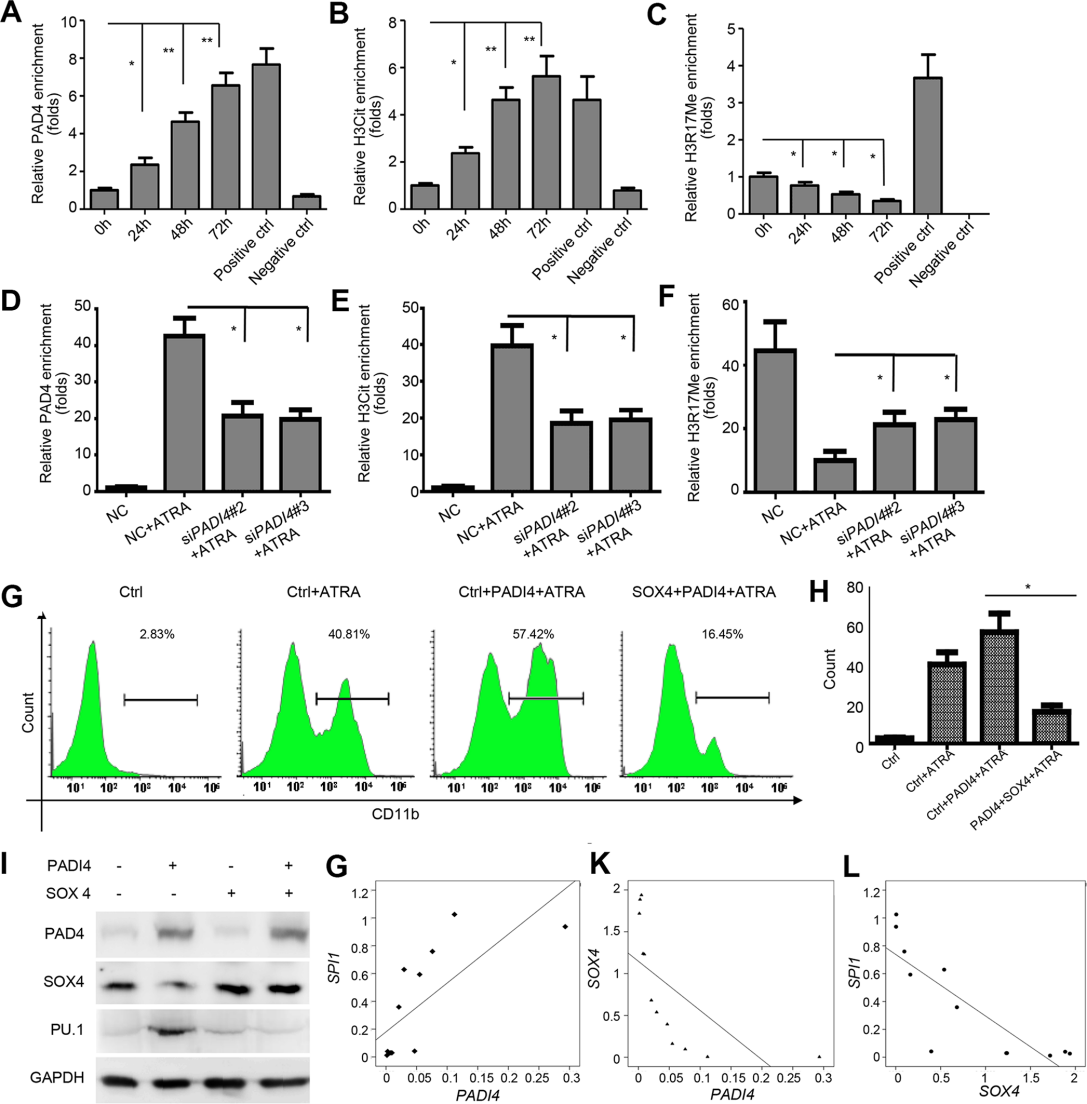
PAD4 regulates SOX4 expression through citrullination and functions in a SOX4-dependent manner **(A–C)** ChIP analysis was applied to detect the binding of PAD4, H3Cit, and H3R17me to the *SOX4* promoter upon ATRA-induced granulocyte differentiation. Positive controls represent the known anti-H3R17Me-positive and anti-H3cit-positive region in the *p21* promoter. Negative control was IgG. (**D–F)** The same assay was also applied to detect the above parameters in the presence of siRNA targeting PAD4. **(G)** and **(H)** FCM analysis of the differentiation of HL-60 after indicated treatment. Ectopic PAD4 or SOX4 expression was induced by electroporation. **(I)** Western blot analysis of protein expression of PAD4, SOX4, and PU.1 in HL-60 cells after electroporation-induced addition of exogenous PAD4, SOX4, or both. (**J–L)** qRT-PCR assays were performed to quantify the expression levels of *PADI4*, *SOX4*, and *SPI1* in clinical samples of APL (*n* = 12). Correlation between *PADI4* and *SOX4* J., *PADI4* and *SPI1* K., as well as *SOX4* and *SPI1* L. by Pearson correlation analysis. *P < 0.05, **P < 0.01. Data are means of biological triplicates (± standard error). All assays were performed with at least three independent preparations and measured in triplicate.

Given that PAD4 can regulate gene expression by citrullinating specific arginine residues on histone H3 and H4 tails [31, 32], thereby antagonizing histone methylation by protein arginine methyltransferases (PRMTs), we explored whether the modification of histone Arg by PAD4 is involved in the transcriptional regulation of SOX4 during ATRA-induced differentiation. To test this, ChIP-qPCR assays were performed to analyze the enrichment of PAD4, as well as histone H3 Arg-17 methylation (H3R17Me) and histone H3 citrullination [CitH3, made against H3 N-terminal peptide (residues 1–20) containing three citrulline residues (Cit2, −8, and −17)] in the *SOX4* promoter of HL-60 cells after ATRA treatment. As expected, more PAD4 bound to the promoter of *SOX4* following ATRA stimulation (Figure 6A). Accordingly, increased histone H3 citrullination (Figure 6B) but a concomitant decrease of histone H3R17 methylation (Figure 6C) occurred over time in the *SOX4* promoter following ATRA treatment. Notably, silencing PAD4 reversed these effects (Figure 6D–6F). Thus, these results suggest that a dynamic chromatin change occurs during the differentiation process and further support the link between PAD4 and the differentiation of leukemia cells.

### SOX4 is required for the activity of PAD4 to promote differentiation

To evaluate the mediation of SOX4 on the activity of PAD4, we used electroporation to re-introduce both PAD4 and SOX4 expression in HL-60 cells in the presence of ATRA. Ectopic expression of PAD4 promoted the differentiation of HL-60 cells, as evidenced by a larger proportion of cells exhibiting CD11b synthesis (Figure 6G and 6H). However, ectopic expression of SOX4 in HL-60 cells attenuated the pro-differentiation effect of overexpressing PAD4 (Figure 6G and 6H). We also investigated whether SOX4 mediated the regulation of PAD4 on PU.1 production. As expected, overexpressed SOX4 in HL-60 cells almost completely blocked the up-regulation of PU.1 that was induced by overexpressing PAD4 (Figure 6I). Collectively, these results confirm that PAD4 suppression in APL results in deregulation of SOX4 and its downstream effector PU.1 to inhibit differentiation, thus forming a functional axis in APL.

### Correlation of the expression levels of PADI4 with SOX4 and SPI1 in clinical samples

Expression levels of *PADI4*, *SOX4*, and *SPI1* were measured to better determine the roles of PAD4 in monocytesfrom APL patients. A significant positive correlation was observed between *PADI4* and *SPI1* (*r*^2^ = 0.733, *P* = 0.007, Figure 6J). However, significant negative correlations were found between *PADI4* and *SOX4* (*r*^2^ = −0.65, *P* = 0.022, Figure 6K) and *SOX4* and *SPI1* (*r*^2^ = −0.834, *P* = 0.001, Figure 6L). These results further support the existence of a PAD4/SOX4/PU.1 axis in APL.

## DISCUSSION

Most cases of APL exhibit the PML-RARα fusion protein and respond to treatment with ATRA or arsenic trioxide [2]. However, the fact that HL-60 cells, which are PML-RARα negative, can be induced to differentiate into more mature granulocytes by ATRA indicates that the degradation of the PML-RARα fusion protein is not the only molecular mechanism to fully or partially differentiate myeloid cells. In this study, to understand the pathological mechanism in APL, microarray analysis was performed in HL-60 cells to comparatively evaluate the differences after ATRA-induced differentiation. Of note, *PADI4* was significantly induced following ATRA exposure when cell differentiation occurred, but was not caused by non-specific stress. To obtain further evidence of the involvement of PAD4 in the abnormal differentiation of APL, we searched the Oncomine database; *PADI4* was found to be down-regulated in APL compared with normal patients (data not shown). Furthermore, the expression of PAD4 in HL-60 cells increased steadily as the exposure time of ATRA went on. These data support the involvement of PAD4 and its dynamic activity in APL.

Previous pathological and genetic studies have demonstrated that PAD4 may be involved in the tumorigenesis and progression from different aspects [14]. Here, we identified that the differentiation of leukemic cells, both PML-RARα positive or negative, could be regulated after modulating the expression or activity of PAD4, suggesting that, besides PML-RARα degradation, an alternative mechanism involving PAD4 may exist to promote differentiation from the promyelocytic stage to mature granulocytes. Furthermore, consistent with previous studies [10, 33], PAD4 was detected in the cytoplasm of HL60 cells before ATRA treatment, indicating its cytoplasmic location may help to maintain the de-differentiation status, through the exact mechanism remain detailed study. In addition, our results demonstrated that ATRA treatment facilitated the nuclear translocation of PAD4, further indicating that PAD4 is needed for ATRA-mediated differentiation. Although induction of *PADI2*, related to *PADI4*, was also observed in our microarray data, its expression change didn't reach the significance level. Thus, PADI4 was selected for the focus of these studies.

Perturbed epigenetic regulation, such as DNA methylation, results in a block of cellular differentiation, as is clinically apparent in APL [34]. Emerging data indicate that the DNMTs, including DNMT1, DNMT3a, and DNMT3b, which catalyze promoter methylation, are upregulated in leukemia [35]. Notably, our results confirm that hypermethylation in the *PADI4* promoter region, which may be catalyzed by DNMT1, contributes its suppression. Furthermore, demethylation has been observed in genes involved in the differentiation of specific cell lines [36]. Consistently, the promoter of *PADI4* undergoes dynamic demethylation during ATRA-induced differentiation, which suggests that *PADI4* acts as a key target for epigenetic refinement of gene expression programs. In addition, hypermethylation of the target genes is reportedly correlated with aggressive/indolent phenotype in AML [37]. Therefore, the relationship between *PADI4* hypermethylation status and AML subtypes needs to be further clarified.

Given the previous findings suggesting a context-specific repressive or activating transcriptional cofactor activity of PAD4 [15], we next focused on clarifying its targets to further understand how PAD4 put its effects on the cell differentiation. Myeloid cell differentiation is controlled by a complex circuitry of lineage-specific transcription factors. PU.1, an ETS family transcription factor, is critical in the determination of the specification of myeloid and common lymphoid progenitors, as well as the development of macrophages and granulocytes [38]. Recently, PU.1 expression was reported to be directly regulated by RUNX1, HSF-1, NF-κB, STAT3, and SOX4 in various diseases [19–23]. Although no direct regulation by PAD4 was observed in our study, interestingly, SOX4 was validated as the direct target of PAD4 and ectopic expression of SOX4 could counteract the activity of PAD4, indicating that a SOX4-dependent manner may exist during PAD4 functions. A previous study showed that SOX4 could block differentiation of myeloid leukemia cells through targeting PU.1 [31], and the regulation of PAD4 towards SOX4 further supports the involvement of PAD4 in the differentiation of leukemia.

It has been reported that PAD4 could convert unmodified or methylated arginine residues at positions H3R2, H3R8, H3R17, and H3R26 to citrulline and counteract the functions of PRMTs [39]. The repressive function of PAD4 has been linked to a negative influence on H3R17 methylation, while arginine methylation at position H3R17 could activate gene expression [34]. We suspected that SOX4 would be susceptible to regulation by PAD4, which acts as a chromatin modulator by citrullinating the promoters of its target genes. Here we show that suppressing the expression or activity of PAD4 led to a decrease in histone citrullination but a concomitant increase in histone Arg methylation at the *SOX4* promoter, and consequentlyactivatedits expression, suggesting that the dynamic modification of histone Arg contributes to the regulation by PAD4 during differentiation. *PADI4* was shown to be transactivated in various solid cancer tissues and could interact with p53 to negatively regulate tumor suppressor genes, such as p21 and OKL38, suggesting that PAD4 functions as an oncogene [11, 26]. These results argue for a context-specific repressive or activating transcriptional cofactor activity of PAD4 if the balance of its activity is shifted between active or repressive arginine methylation, which needs further study in granulocyte differentiation.

**Figure 7:**
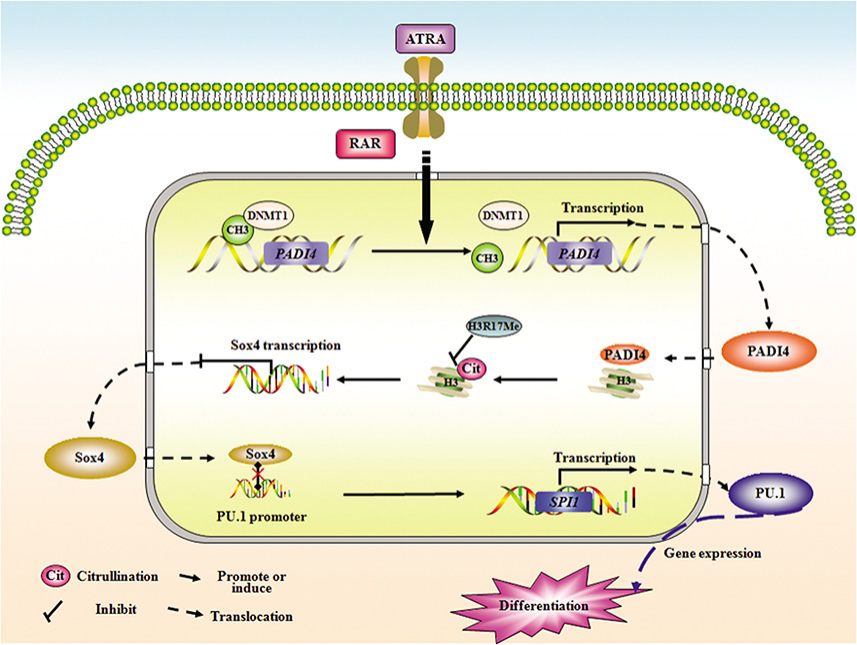
A putative model illustrating the role of PAD4 in the abnormal differentiation of APL cells Demethylation in *PADI4* and a simultaneous restoration of its expression occurred during ATRA-induced differentiation. Furthermore, ATRA stimulation facilitates the nuclear translocation of PAD4, and in turn, PAD4could promote the differentiation of APL cells in the presence of ATRA. Mechanistically, PAD4 could directly regulate SOX4 expression via citrullinating histone3, thereby antagonizing the methylation of H3Arg17. In addition, PU.1 is validated as the direct target of SOX4, and PAD4 could function to promote differentiation or regulate PU.1 expression in a SOX4-dependent manner, suggesting a functional pathway may form among PAD4, SOX4, and PU.1 in the pathological mechanism of APL.

Collectively, these results demonstrate that suppression of PAD4 is an important mechanism leading to the maturation block at the promyelocytic stage seen in human APL. Although gene microarray analysis is still needed to fully screen the target genes of PAD4, our study confirms the formation of a functional axis among PAD4, SOX4, and PU.1, which may also represent a promising path for interfering with the abnormal differentiation of leukemia cells (Figure 7).

## MATERIALS AND METHODS

### Materials

ATRA (All-trans-retinoic acid), 5-aza-2-deoxycytidine (DAC), trichostatin A (TSA) and anti-PADI4 (P4874) were purchased from Sigma (St. Louis, MO). Cl-amidine was from Cayman Chemical Company in USA. pGL3-basic vector and Dual Luciferase Reporter Assay System were from Promega Corp. Antibody specific to SOX4 (ab80261), H3R17Me (Ab8284), H3Cit (Ab5103), DNMT1 (ab13537), DNMT3a (ab2850), DNMT3b (ab2851), Tubulin (ab126165), Lambin B (ab16048) and horseradish peroxidase coupled secondary antibodies were obtained from Abcam (Cambridge, MA, USA). Anti-GAPDH (sc-47724) and anti-PU.1 antibody (sc-365208) were from Santa Cruz Biotechnology Anti-human CD11b antibody (11–0113) was from eBiosciences in San Diego of USA.

### Patients and cell culture

HL-60 and NB4 cell line were obtained from ATCC (Manassas, VA). The cells were cultured at 37°C under 5% CO_2_ in 1640 medium supplemented with 10% FBS (Invitrogen-Gibco), 100 U/ml penicillin, and 100 μg/ml streptomycin. Bone marrow and peripheral blood leukemicblood samples were collected after informed consent from healthy and 14 patients with newly diagnosed AML. The human monocytes were prepared by Ficoll Hypaque (Nygaard) gradient centrifugation and then cryopreserved or cultured.

### ATRA treatment and microarray analysis

Leukemic cells were treated with 1 μM ATRA for 72 h. For microarray analysis, HL-60 cells were treated with ATRA for 72 hours to induce differentiation into granulocytes and the total RNA was extracted using Trizol reagent. Then, the Affymetrix GeneChip Human Genome U133 Plus 2.0 Array was performed at Capital Bio in Beijing.

### Immunofluorescence staining and microscopy

2 × 10^5^ HL-60 cells were centrifuged onto slides and were fixed for 10 min with 4% PFA, followed by permeabilization for 20 min at room temperature with 0.5% Triton X-100. After incubation for blockade of nonspecific binding for 30 min, PADI4 primary antibodies were added for incubation for 2 h at room temperature. Samples were further stained with suitable Alexa Fluor-conjugated secondary antibodies (Thermo Fisher Scientific, PA, USA). A series of control samples were prepared as follows: (1) the samples were only incubated with the primary antibodies, (2) thesamples were only incubated with the secondary antibodies or (3) the samples were incubated with normal rabbit serum. The images were acquired on an Olympus IX71 fluorescence microscope (Olympus Co., Tokyo, Japan). DAPI (4′, 6′-diamidino-2-phenylindole hydrochloride; Molecular Probes, Invitrogen) was used to stain nuclei.

### RNA isolation and real time quantitative PCR (qRT-PCR)

Total RNA was extracted with TRIzol reagent according to the manufacturer's instructions (Invitrogen, CA). The concentration, quality, and purity of RNA were assessed with the use of the RNA 6000 Nano assay on the Agilent 2100 Bioanalyzer (Agilent). None of the samples showed RNA degradation (ratio of 28S ribosomal RNA to 18S ribosomal RNA of at least 2) or contamination by DNA. For reverse transcription, samples were incubated in an Eppendorf PCR system at 42°C for 30 min, then at 90°C for 5 min and at 5°C for 5 min. Reverse transcription mixtures were subjected to qPCR with specific primers as shown in Supplementary Table 1. PCR was performed in a 10 μl total volume that contained 1 μl of cDNA, 5 μl of SYBR Green Real-time PCR Master Mix (ToYoBo, Japan) and 1 μl of each primer. PCR was conducted with the following conditions: 10 s at 95°C; 40 cycles of 5 s at 60°C and 10 s at 72°C; 30 s at 65°C. Melting analysis of the PCR products was conducted to validate the amplification of the specific product and the results were normalized to *GAPDH*.

### Western blot

Cells were lysed with M-PER Protein Extraction reagent (Pierce, Rockford, IL) supplemented with a protease inhibitor mixture. The nuclear proteins were extracted using Nuclear and Cytoplasmic Extraction Reagents kits (Thermo scientific, USA). Protein concentrations in the extracts were measured with a bicinchoninic acid assay (Pierce, Rockford, IL). Equal amounts of extracts were separated by SDS-PAGE and transferred onto PVDF membrane for immunoblot analysis. The membranes were blocked with 5% skimmed milk in TBST for 1 hour and incubated with primary antibodies at 1:1000 dilutions overnight at 4°C. After HRP-conjugated secondary antibody was added, proteins were detected using an ECL kit.

### Construction of *SOX4* luciferase reporter plasmids

The human *SOX4* gene promoter fragments nt −2296 to +153, −2032 to +153, −1289 to +153, −840 to +153, −489 to +153, were amplified by PCR using human genomic DNA from HL-60 leukemia cells as template and inserted into the SAC I and BGL II sites of the luciferase reporter plasmid pGL3-basic vector, yielding the reporter constructs SOX4(P1), SOX4 (P2), SOX4 (P3), SOX4 (P4), SOX4 (P5), respectively. The SOX4 promoter fragments nt-2296 to −489 were amplified by PCR and inserted into the SAC I and BGL II sites of pGL3-basic vector, yielding the reporter constructs SOX4 (P6). All constructs were confirmed by DNA sequencing. The sequences of primers used are shown in Supplementary Table 1.

### Transfection and luciferase assay

Transfection of HL-60 cells were electroporated with a BTX ECM 830 square wave electroporator (BTX, San Diego, CA) with three 225V pulses of 8 ms pulse length and waiting for 1s between pulses. After the pulse, the cells were transferred to RPMI 1640 medium supplemented with 10% fetal bovine serum. Cell viability of the electroporated cells was more than 90% after counting by trypan blue exclusion assay. For transient silencing by duplexes of small interfering RNA into HL-60 cells, HiPerFect Transfection Reagent was used (Qiagen, Hilden, Germany). Target sequences for transient silencing were 5′-GCACGUCCUUCAGCAUCAATTUUGAUGCUGAAGGACGUGCTT-3′ for siPADI4#1, 5′-CCGGUGGAAAGCACAACAUTTAUUCACAGCUCUGGUUGGCTT-3′ for siPADI4#2, 5′-GCCAACCAGAGCUGUGAAATTUUGAUGCUGAAGGACGUGCTT-3′ for siPADI4#3 and 5′-GGACAGACGAAGAGUUUAATT-3′ (sense), 5′-UUAAACUCUUCGUCUGUCCTT-3′ (anti-sense) for SOX4, scrambled control sequences were 5′-UUCUCCGAACGUGUCACGUUUCUCCGAACGUGUCACGU-3′. HEK293 cell was co-transfected with the mixture of the indicated luciferase reporter plasmid and pRL-TK-Renilla luciferase plasmid using lipofectamine 2000 transfection reagent (Invitrogen). 24 h later, luciferase activities were measured with a Dual Luciferase Reporter Assay System, according to the manufacturer's instructions. Data are normalized for transfection efficiency by dividing firefly luciferase activity with that of Renilla luciferase.

### Chromatin immunoprecipitation quantitative PCR (ChIP-qPCR) assay

Chromatin from leukemia cells was fixed and immunoprecipitated using the ChIP assay kit as recommended by the manufacturer (Upstate Biotechnology, NY). The purified chromatin was immunoprecipitated using 3 μg of anti-PADI4, anti-DNMT1, anti-H3R17Me, anti-H3Cit, or irrelevant antibody (IgG). After DNA purification, the presence of the selected DNA sequence was assessed by qPCR. The primers of different regions for *SOX4/SPI1* promoter were shown in Supplementary Table 1. PCR products were separated and visualized as described above. The average size of the sonicated DNA fragments subjected to immunoprecipitation was 500 bp as determined by ethidium bromide gel electrophoresis. The qPCR primers to detect the binding of PADI4, H3cit and H3R17Me to the *SOX4* promoter were forward 5′-AGC AGGCTGTGGCTCTGATT-3′ and reverse 5′-CAAAAT AGCCACCAGCCTCTTCT-3′, and DNMT1 were forward 5′-CATTGACACCCATCTAGA-3′ and reverse 5′-CTGGG CTGGTTCCTTTATAT-3′.

### Bisulfite conversion of DNA samples and MSP

Bisulfite conversion was carried out using reagents provided in EpiTect Bisulfite Kit (Qiagen 59104). 1 μg of DNA was treated with sodium bisulfite following the manufacturers' recommendations. Following conversion, the bisulfite-converted DNA was resuspended in a total volume of 20 μl. Methylation specific primers for PADI4 promoters were designed using MethPrimer program. Methylation specific quantitative PCR primers were designed using the MethPrimer tool. M primer: 5′-ATATATGGGTATTTTGATAGGACGT-3′ (sense), 5′-T AACGTAAACATAAAACGTTTCGTA-3′ (anti-sense); U primer: 5′-ATATATGGGTATTTTGATAGGATGT-3′ (sense), 5′-TAACATAAACATAAAACATTTCATA-3′ (anti-sense). Bisulfite converted genomic DNA was PCR amplified using methylation specific primers.

### Methylation DNA immunoprecipitation (MeDIP) analysis

The MeDIP analysis was carried out using MagMeDIP Kit (Diagenode, Denville, NJ). Briefly, after sonication to shear, the fragmented DNA was immunoprecipitated with anti-methylcytosine antibody at 4°C overnight. Then, the pulled-down DNA on magnetic beads were washed and digested with proteinase K and isolated from beads. The primer set, sense 5′-ACTGGTACCAGCATTGAC-3′ and anti-sense 5′-TAGGAAGCCCCTGGGCTGGT-3′, which covers the DNA sequence of the CpGs of human PADI4 was used for qPCR assays. For qPCR, the enrichment of MeDIP DNA was calculated as described before [40] and the relative methylated DNA ratios were then normalized based on the control as 100% of methylated DNA.

### Flow cytometry (FCM)

Control and HL-60 cells treated with ATRA (1×10^6^ cells) were washed with PBS containing 1% FCS and 0.01% sodium azide were incubated for 30 min in FCS at 4°C. Subsequently, FITC-conjugated anti-human CD11b antibody was added to the cells and incubated at 25°C for 45 min followed by washing with PBS. The cells were then fixed in 1% paraformaldehyde and analyzed on FACSVerse (BD Biosciences Pharmingen) Flow cytometer. Isotypic rat IgG was also used to check for nonspecific binding.

### Statistical analysis

All data are presented as means ± S.D. of three or four experiments. Analysis was performed using a Student's *t* test. Values of *P* < 0.05 were considered significant.

## SUPPLEMENTARY FIGURES AND TABLES



## References

[1] Fialkow PJ, Janssen JW, Bartram CR (1991). Clonal remissions in acute nonlymphocytic leukemia: evidence for a multistep pathogenesis of the malignancy. Blood.

[2] Nasr R, Guillemin MC, Ferhi O, Soilihi H, Peres L, Berthier C, Rousselot P, Robledo-Sarmiento M, Lallemand-Breitenbach V, Gourmel B, Vitoux D, Pandolfi PP, Rochette-Egly C (2008). Eradication of acute promyelocytic leukemia-initiating cells through PML-RARA degradation. Nat Med.

[3] Lallemand-Breitenbach V, Zhu J, Chen Z, de The H (2012). Curing APL through PML/RARA degradation by As2O3. Trends Mol Med.

[4] Imran M, Park TJ, Lim IK (2012). TIS21/BTG2/PC3 enhances downregulation of c-Myc during differentiation of HL-60 cells by activating Erk1/2 and inhibiting Akt in response to all-trans-retinoic acid. Eur J Cancer.

[5] Yang J, Ikezoe T, Nishioka C, Yokoyama A (2013). Over-expression of Mcl-1 impairs the ability of ATRA to induce growth arrest and differentiation in acute promyelocytic leukemia cells. Apoptosis.

[6] Nakashima K, Hagiwara T, Ishigami A, Nagata S, Asaga H, Kuramoto M, Senshu T, Yamada M (1999). Molecular characterization of peptidylarginine deiminase in HL-60 cells induced by retinoic acid and 1alpha, 25-dihydroxyvitamin D (3). J Biol Chem.

[7] Nakashima K, Arai S, Suzuki A, Nariai Y, Urano T, Nakayama M, Ohara O, Yamamura K, Yamamoto K, Miyazaki T (2013). PAD4 regulates proliferation of multipotent haematopoietic cells by controlling c-myc expression. Nat Commun.

[8] Zavala-Cerna MG, Gonzalez-Montoya NG, Nava A, Gamez-Nava JI, Moran-Moguel MC, Rosales-Gomez RC, Gutierrez-Rubio SA, Sanchez-Corona J, Gonzalez-Lopez L, Davalos-Rodriguez IP, Salazar-Paramo M (2013). PADI4 haplotypes in association with RA Mexican patients, a new prospect for antigen modulation. Clin Dev Immunol.

[9] Christophorou MA, Castelo-Branco G, Halley-Stott RP, Oliveira CS, Loos R, Radzisheuskaya A, Mowen KA, Bertone P, Silva JC, Zernicka-Goetz M, Nielsen ML, Gurdon JB, Kouzarides T (2014). Citrullination regulates pluripotency and histone H1 binding to chromatin. Nature.

[10] Chang X, Han J (2006). Expression of peptidylarginine deiminase type 4 (PAD4) in various tumors. Mol Carcinog.

[11] Yao H, Li P, Venters BJ, Zheng S, Thompson PR, Pugh BF, Wang Y (2008). Histone Arg modifications and p53 regulate the expression of OKL38, a mediator of apoptosis. J Biol Chem.

[12] Liu GY, Liao YF, Chang WH, Liu CC, Hsieh MC, Hsu PC, Tsay GJ, Hung HC (2006). Overexpression of peptidylarginine deiminase IV features in apoptosis of haematopoietic cells. Apoptosis.

[13] Rohde C, Schoofs T, Muller-Tidow C (2013). The limited contribution of DNA methylation to PML-RARalpha induced leukemia. Oncotarget.

[14] Kolodziej S, Kuvardina ON, Oellerich T, Herglotz J, Backert I, Kohrs N, Buscató El, Wittmann SK, Salinas-Riester G, Bonig H, Karas M, Serve H, Proschak E (2014). PADI4 acts as a coactivator of Tal1 by counteracting repressive histone arginine methylation. Nature communications.

[15] Luo Y, Arita K, Bhatia M, Knuckley B, Lee YH, Stallcup MR, Sato M, Thompson PR (2006). Inhibitors and inactivators of protein arginine deiminase 4: functional and structural characterization. Biochemistry.

[16] Fischbach NA, Rozenfeld S, Shen W, Fong S, Chrobak D, Ginzinger D, Kogan SC, Radhakrishnan A, Le Beau MM, Largman C, Lawrence HJ (2005). HOXB6 overexpression in murine bone marrow immortalizes a myelomonocytic precursor *in vitro* and causes hematopoietic stem cell expansion and acute myeloid leukemia *in vivo*. Blood.

[17] Collins C, Wang J, Miao H, Bronstein J, Nawer H, Xu T, Figueroa M, Muntean AG, Hess JL (2014). C/EBPalpha is an essential collaborator in Hoxa9/Meis1-mediated leukemogenesis. Proc Natl Acad Sci U S A.

[18] Goldberg L, Tijssen MR, Birger Y, Hannah RL, Kinston SJ, Schütte J, Beck D, Knezevic K, Schiby G, Jacob-Hirsch J, Biran A, Kloog Y, Marcucci G (2013). Genome-scale expression and transcription factor binding profiles reveal therapeutic targets in transgenic ERG myeloid leukemia. Blood.

[19] Lyu X, Xin Y, Mi R, Ding J, Wang X, Hu J, Fan R, Wei X, Song Y, Zhao RY (2014). Overexpression of Wilms tumor 1 gene as a negative prognostic indicator in acute myeloid leukemia. PloS one.

[20] Lugthart S, van Drunen E, van Norden Y, van Hoven A, Erpelinck CA, Valk PJ, Beverloo HB, Lowenberg B, Delwel R (2008). High EVI1 levels predict adverse outcome in acute myeloid leukemia: prevalence of EVI1 overexpression and chromosome 3q26 abnormalities underestimated. Blood.

[21] Scott EW, Simon MC, Anastasi J, Singh H (1994). Requirement of transcription factor PU. 1 in the development of multiple hematopoietic lineages. Science.

[22] Hughes JM, Legnini I, Salvatori B, Masciarelli S, Marchioni M, Fazi F, Morlando M, Bozzoni I, Fatica A (2015). C/EBPalpha-p30 protein induces expression of the oncogenic long non-coding RNA UCA1 in acute myeloid leukemia. Oncotarget.

[23] Coudé MM, Braun T, Berrou J, Dupont M, Bertrand S, Masse A, Raffoux E, Itzykson R, Delord M, Riveiro ME, Herait P, Baruchel A, Dombret H (2015). BET inhibitor OTX015 targets BRD2 and BRD4 and decreases c-MYC in acute leukemia cells. Oncotarget.

[24] DeKoter RP, Walsh JC, Singh H (1998). PU. 1 regulates both cytokine-dependent proliferation and differentiation of granulocyte/macrophage progenitors. EMBO J.

[25] Li P, Yao H, Zhang Z, Li M, Luo Y, Thompson PR, Gilmour DS, Wang Y (2008). Regulation of p53 target gene expression by peptidylarginine deiminase 4. Mol Cell Biol.

[26] Staber PB, Zhang P, Ye M, Welner RS, Levantini E, Di Ruscio A, Ebralidze AK, Bach C, Zhang H, Zhang J, Vanura K, Delwel R, Yang H (2014). The Runx-PU. 1 pathway preserves normal and AML/ETO9a leukemic stem cells. Blood.

[27] Jego G, Lanneau D, De Thonel A, Berthenet K, Hazoumé A, Droin N, Hamman A, Girodon F, Bellaye PS, Wettstein G, Jacquel A, Duplomb L, Le Mouël A (2014). Dual regulation of SPI1/PU. 1 transcription factor by heat shock factor 1 (HSF1) during macrophage differentiation of monocytes. Leukemia.

[28] Bonadies N, Neururer C, Steege A, Vallabhapurapu S, Pabst T, Mueller BU (2010). PU. 1 is regulated by NF-kappaB through a novel binding site in a 17 kb upstream enhancer element. Oncogene.

[29] Hegde S, Ni S, He S, Yoon D, Feng GS, Watowich SS, Paulson RF, Hankey PA (2009). Stat3 promotes the development of erythroleukemia by inducing Pu. 1 expression and inhibiting erythroid differentiation. Oncogene.

[30] Aue G, Du Y, Cleveland SM, Smith SB, Davé UP, Liu D, Weniger MA, Metais JY, Jenkins NA, Copeland NG, Dunbar CE (2011). Sox4 cooperates with PU. 1 haploinsufficiency in murine myeloid leukemia. Blood.

[31] Wang Y, Wysocka J, Sayegh J, Lee YH, Perlin JR, Leonelli L, Sonbuchner LS, McDonald CH, Cook RG, Dou Y, Roeder RG, Clarke S, Stallcup MR (2004). Human PAD4 regulates histone arginine methylation levels via demethylimination. Science.

[32] Tanikawa C, Espinosa M, Suzuki A, Masuda K, Yamamoto K, Tsuchiya E, Ueda K, Daigo Y, Nakamura Y, Matsuda K (2012). Regulation of histone modification and chromatin structure by the p53-PADI4 pathway. Nat Commun.

[33] Wang L, Chang X, Yuan G, Zhao Y, Wang P (2010). Expression of peptidylarginine deiminase type 4 in ovarian tumors. Int J Biol Sci.

[34] Arteaga MF, Mikesch JH, Fung TK, So CW (2015). Epigenetics in acute promyelocytic leukaemia pathogenesis and treatment response: a TRAnsition to targeted therapies. Br J Cancer.

[35] Mizuno S, Chijiwa T, Okamura T, Akashi K, Fukumaki Y, Niho Y, Sasaki H (2001). Expression of DNA methyltransferases DNMT1, 3A, and 3B in normal hematopoiesis and in acute and chronic myelogenous leukemia. Blood.

[36] Wang E, Kawaoka S, Yu M, Shi J, Ni T, Yang W, Zhu J, Roeder RG, Vakoc CR (2013). Histone H2B ubiquitin ligase RNF20 is required for MLL-rearranged leukemia. Proc Natl Acad Sci U S A.

[37] Bartholdy B, Christopeit M, Will B, Mo Y, Barreyro L, Yu Y, Bhagat TD, Okoye-Okafor UC, Todorova TI, Greally JM, Levine RL, Melnick A, Verma A (2014). HSC commitment-associated epigenetic signature is prognostic in acute myeloid leukemia. J Clin Invest.

[38] Karpurapu M, Ranjan R, Deng J, Chung S, Lee YG, Xiao L, Nirujogi TS, Jacobson JR, Park GY, Christman JW (2014). Kruppel like factor 4 promoter undergoes active demethylation during monocyte/macrophage differentiation. PloS one.

[39] Cuthbert GL, Daujat S, Snowden AW, Erdjument-Bromage H, Hagiwara T, Yamada M, Schneider R, Gregory PD, Tempst P, Bannister AJ, Kouzarides T (2004). Histone deimination antagonizes arginine methylation. Cell.

[40] Zhang C, Su ZY, Khor TO, Shu L, Kong AN (2013). Sulforaphane enhances Nrf2 expression in prostate cancer TRAMP C1 cells through epigenetic regulation. Biochem Pharmacol.

