# Supplementary material for: A novel PAD4/SOX4/PU.1 signaling pathway is involved in the committed differentiation of acute promyelocytic leukemia cells into granulocytic cells
Source: Oncotarget. 2015 Dec 10;7(3):3144–57. doi: 10.18632/oncotarget.6551 (PMC4823096; doi:10.18632/oncotarget.6551)
Supplement: Supplementary file 1 [file oncotarget-07-3144-s001.pdf]

A novel PAD4/SOX4/PU.1 signaling pathway is involved in the committed differentiation of acute promyelocytic leukemia cells into granulocytic cells

Supplementary Materials

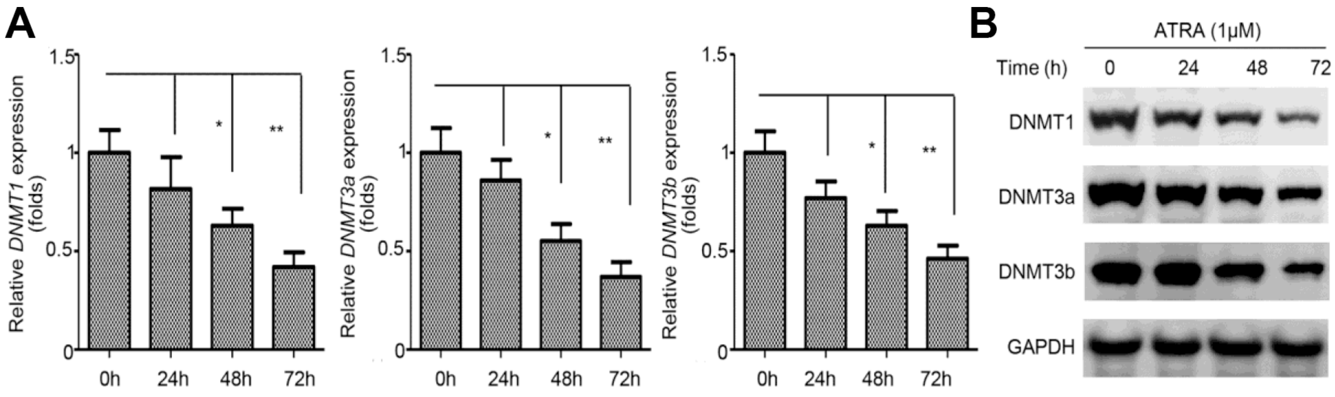

Supplementary Figure S1: ATRA treatment suppresses the expression of DNMTs. (**A**) and (**B**) Expression of three DNMTs was detected by RT-PCR (A) and Western blot analysis (B) after treatment with ATRA (1 μM) at indicated timepoints.

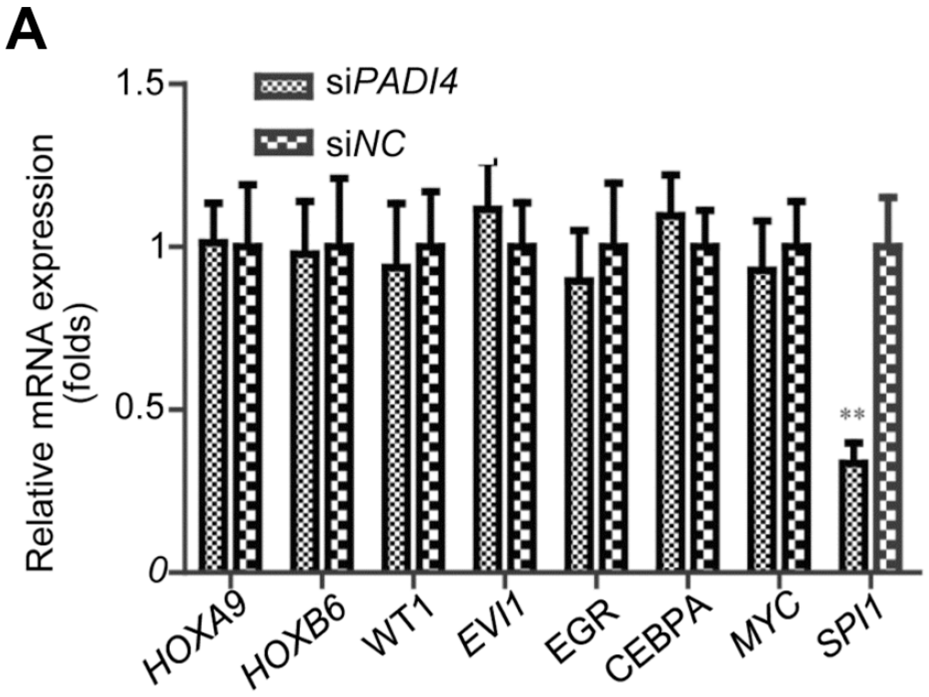

Supplementary Figure 2: PU.1 was regulated by PAD4. (**A**) Expression of *SPI1, HOXA9, HOXB6, WT1, EVI1,* and *EGR* after silencing PAD4 in ATRA-induced HL-60 cells. $^{**}P < 0.01$. Data are means of biological triplicates (± standard error) and representative of triplicate experiments.

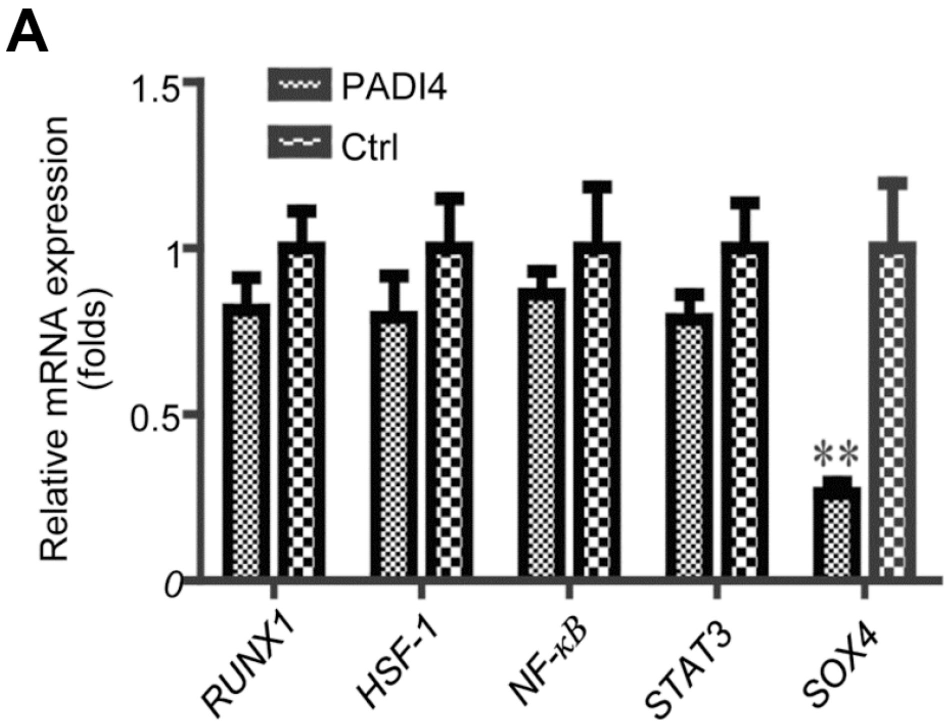

Supplementary Figure 3: SOX4 was regulated by PAD4. (A) Expression of *RUNX1, HSF-1, NF*-κB*, STAT3*, and *SOX4* after silencing PAD4 in ATRA-induced HL-60 cells. $^{**}P < 0.01$. Data are means of biological triplicates (± standard error) and representative of triplicate experiments.

Supplementary Table S1: Primers used in this study

Gene	Forward	Reverse
**qRT-PCR**		
PADI4	GTTTAGGGTCAGACAGTCCTGG	AGATGTGAGTAGTGGCACATGC
SOX4	ACACTGGTGGCAGGTTAAGG	CGCTGTTTGGATTTCCTGAT
PU.1	CTGTTGGACCTGCTCCGCA	TGCGGAGCAGGTCCAACAG
GAPDH	AATGGGCAGCCGTTAGGAAA	GCGCCCAATACGACCAAATC
RUNX1	GGACGCCAGAAGGAAGTCAA	TCGCAGCCAGGAAAGAAGTT
HSF-1	CCGTGGACACCCTCTTGTC	GAGCTCATTCTTGTCCAGGCA
STAT3	CTGCCCCATACCTGAAGACC	TCCTCACATGGGGGAGGTAG
NF-κB	CCAACAGATGGCCCATACCT	AACCTTTGCTGGTCCCACAT
**ChIP**		
PU.1–1	GATGGATGGGTAGATGAGTG	CAGCCATCCACAAATCCACC
PU.1–2	TGGATGGCTGGATGAATGAG	ATGCAGTATCTTTTTGGTAT
PU.1–3	ACTGCATTTGTATGTTTATC	CCAATTCCATCCATGTTGC
PU.1–4	GATACTGCATTTGTATGTTT	GATAATGTCCTCCAATTCC
PU.1–5	AGGACATTATCATATGCGAA	CATTCATTCATCCATCCATC
PU.1–6	TGAATGAATGAAGGGGTAGG	CAGTCCATCAGTCCACAA
PU.1–7	GTGGACTGATGGACTGATAG	CCCATGCATCTATTTGTC
PU.1–8	GACAGACAAATAGATGCATG	CCTAGGGCTCTGTTTCCAGCC
PU.1–9	ATAGATGCATGGGAAAACAG	TGGGTTCTTATACCCTCC
PU.1–10	AGCAGCACTATGCTGAAGAC	GCTCTAACCCAACAAATGC
PU.1–11	TAGAGCAAAAGCCTCCCAGT	AACCCGTTTGCATAAATCTCT
PU.1–12	ACGGGTTGGGGCGGTGATGT	AATGCAGAGCCCCTCAGGATG
SOX4-I	TTAAAGAGCGTGCAAGAA	AGCCAAGACTGTGAAAGG
SOX4-II	CAGTATTTCACCCTTACCC	GATTTCTGCTGCCTCTTT
SOX4-III	TACGGAGCACTACCTAAT	GTAAATCCCTTCAGAACC
SOX4-IV	CCCTTCGATTCAAGTAAC	TAGGCAGATTTCCAGAGT
SOX4-V	ACTTTATAGAGGGTTGTTGT	GTCCTGCCTGTTATTACTT
**Plasmid**		
pGL3- SOX4 (P1)	CGAGCTCAGGTGCCTGTGTGTTT	GGAAGATCTTCCGCCTCGCGCCTCTT
pGL3- SOX4 (P2)	CGAGCTCCAATGGAATGGCAGGGT	GGAAGATCTTCCGCCTCGCGCCTCTT
pGL3- SOX4 (P3)	CGAGCTCACAATACATCAGGTGC	GGAAGATCTTCCGCCTCGCGCCTCTT
pGL3- SOX4 (P4)	CGAGCTCGAGCACTACCTAATGTG	GGAAGATCTTCCGCCTCGCGCCTCTT
pGL3- SOX4 (P5)	CGAGCTCTGCACCAGAGGCTGATT	GGAAGATCTTCCGCCTCGCGCCTCTT
pGL3- SOX4 (P6)	CGAGCTCAGGTGCCTGTGTGTTT	GGAAGATCTAATCAGCCTCTGGTGCA
